# High-efficiency terahertz-wave generation based on extended interaction oscillator with strongly-coupled 2π mode operation

**DOI:** 10.1038/s41598-024-66037-6

**Published:** 2024-07-02

**Authors:** Che Xu, Jiani Lu, Yongliang Tang, Xianfeng Tang

**Affiliations:** School of Physical Science and Technology, Southwest Jiaotong University, Chengdu, 610031 China

**Keywords:** Extended interaction oscillator (EIO), Terahertz sources, Vacuum electronics, Strongly-coupled 2π mode, Electrical and electronic engineering, Plasma physics, Electronics, photonics and device physics, Electronic and spintronic devices, Applied physics

## Abstract

A slow-wave structure improvement for enhancing the 2π-mode electronic efficiency is embodied in the validation of an extended interaction oscillator (EIO), which has an electronic efficiency of 6.52% at 0.22 THz from particle-in-cell (PIC) calculations. A 2π-mode bi-periodic slow-wave structure (BPSWS) with staggered long and short slots is utilized for optimizing the circuit performance. The proposed BPSWS has the capability of combining the respective advantages for both π and 2π-mode in terms of coupling performance and output performance, thus supporting a strongly-coupled 2π-mode with higher coupling capability. Compared with the typical mono-periodic SWS (MPSWS), the adopted strongly-coupled 2π-mode effectively improves the characteristic impedance M^2^R/Q by 103% to 66.79 Ω, the coupling coefficient by 66% to 0.497, and the normalized wave-amplitude by 22%. Accordingly, 503 W of average output power can be derived for the BPSWS-EIO with a 25.7 kV and 0.3 A sheet beam injected. Cold-test experiments were conducted, confirming that the 0.22 THz structure exhibits commendable fabrication precision and consistency and thus demonstrates the expected frequency response.

## Introduction

Teraheriz vacuum electronic devices (VEDs) are recognized as potent high-frequency radiation sources, supporting higher power and electronic efficiency than lasers or semiconductor sources^1–4^. Among THz VEDs, the Extended Interaction Devices (EIDs) manifest their superiority through compact and efficient performance characteristics, presenting enormous potential for high-resolution imaging, high data rate communications, diagnostic systems, and space applications. To date, EIDs, including Extended Interaction Oscillator (EIO) and Extended Interaction Amplifier (EIA), have demonstrated their capabilities^2^ from 17 to 280 GHz, with the capability approaching 1 THz.

Although significant terahertz radiation power can be generated through methods based on cyclotron radiation and traveling wave amplification effects, these methodologies come with constraints; the former imposes strict requirements on the strength and spatial distribution of the magnetic field^5^, whereas the latter typically requires employing numerous periodic beam-wave interaction structures to achieve sufficient gain, generally more than 50 periods^6,7^. Systems are even more massive in response to these factors, thereby imposing certain limitations on the practical progress for terahertz VEDs.

To fully utilize the performance advantages of traveling wave tubes and multi-cavity klystrons, EIO and EIA have integrated the characteristics of slow-wave structures (SWSs) and resonant cavities to strive for sufficient gain within a certain bandwidth and length. The gain of the resonant slow-wave structure in the EID can be represented by the characteristic impedance *R/Q* or the more advanced term, effective characteristic impedance *M*^*2*^*R/Q*^8^. Especially when operating in standing wave mode (π or 2π mode), the *R/Q* value of the resonator can be maximized, where *M* represents the coupling coefficient.

The establishment of a simple, easily manufacturable SWS that supports operation modes with high coupling coefficients and high characteristic impedances has been a hot topic in THz-EID research. In recent years EID research projects in the high frequency range have tended to move towards higher-order modes both transversely (TM_n1_ modes) and longitudinally (π modes), based on the more mature, traditional 2π mode. Extensive results show that the TM_n1_ mode offers a larger longitudinal dimension that facilitates the manufacture of terahertz systems^9–11^, while the π mode is increasingly adopted in many EID technologies due to its superior resonator coupling capability^12–14^. The measure of power level, as determined by the ratio of power to frequency squared (*Pf*^*2*^ (GW·GHz^2^)), indicates that SWS operating in the π mode has achieved reliable physical performance at this stage. For instance, a 0.22 THz π-mode bi-periodic EIA, with a cavity *M*^*2*^*R/Q* of 44 Ω, exhibits a *Pf*^*2*^ value of 0.032, 660 W average power, and 2% efficiency^14^; a 94 GHz π-mode bi-periodic EIO, operating at an *R/Q* of 346 Ω, achieves a *Pf*^*2*^ value of 0.0052 (592 W average power), with an efficiency of 6.6%^12^. However, problems are encountered in practical studies where the π-mode has difficulty extracting power longitudinally in the over-dimensional TM_n1_-mode state, thus limiting the saturation output power^15^ (primarily due to its excessive external quality factor *Q*_*e*_). In contrast, the 2π mode offers reliable output power, leveraging its advantage of smaller *Q*_*e*_. For example, a periodic cusp magnet equipped, 94.9 GHz mono-periodic EIO has demonstrated a *Pf*^*2*^ value of 0.092 and a peak power of 10.2 kW^16^; a 94 GHz mono-periodic EIA with a lower loaded-quality-factor *Q*_*l*_ of 210 achieves a peak power of 7.7 kW, with an efficiency of 8.6% and a *Pf*^*2*^ value of 0.068^4^. Alternatively, if the much-proven optional 2π mode is reconsidered it faces the restriction of *M*^*2*^*R/Q* than π mode^15,17^. The evolution and development of THz-EID technology have been full of trade-offs and compromises due to the respective advantages for the 2π and π modes.

**Figure 1:**
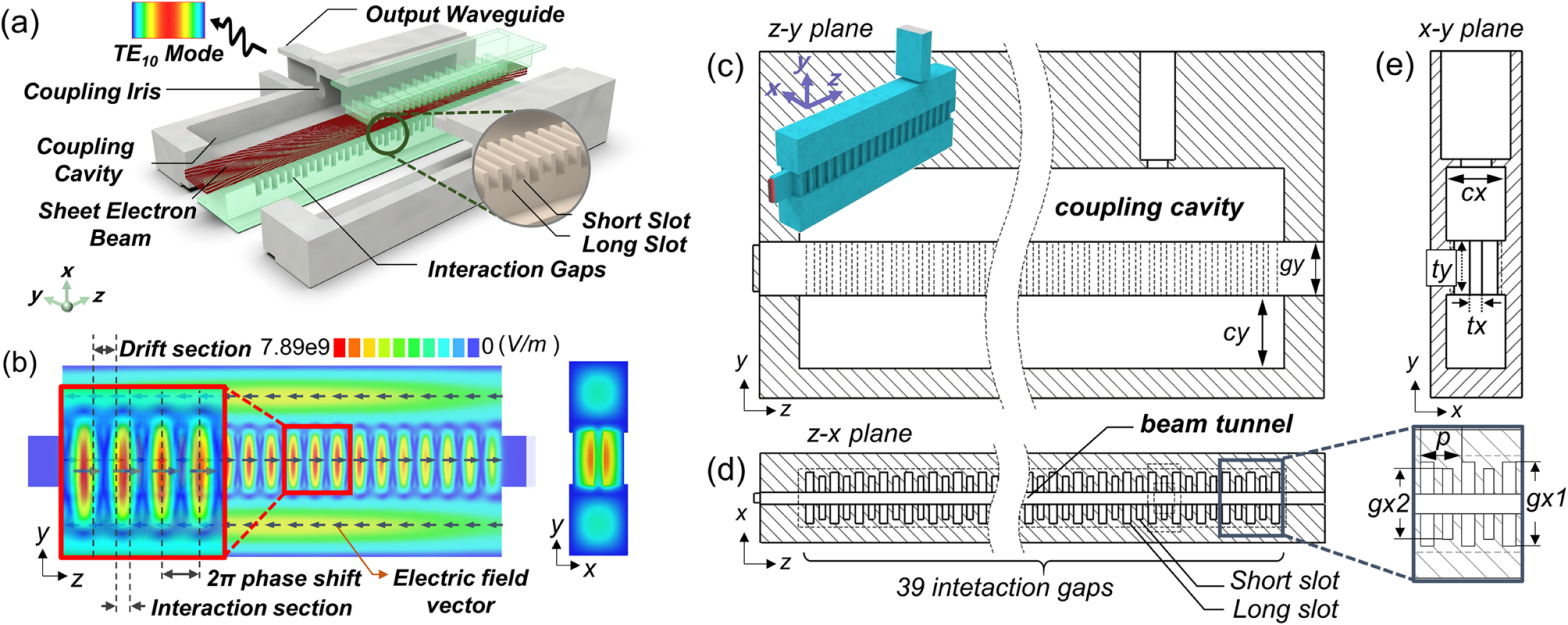
(**a**) The three-dimentional schematic diagram of the proposed slow-wave structure. (**b**) The electric field distribution in the y–z plane, and the structural profiles of the cross-section in (**c**) y–z plane, (**d**) x–z plane, and (**e**) x–y plane and for the BPSWS.

In an effort to enhance the effective characteristic impedance of the 2π mode and to improve inter-gap coupling performance, this study integrates the relative advantages for the π and 2π modes. This approach achieves a balance between the coupling efficiency and output capability of the device, termed as the ‘strongly-coupled 2π mode’ within this paper. The newly developed strongly-coupled 2π mode BPSWS merges the strengths of the previously mentioned two standing-wave modes, yielding a higher effective characteristic impedance and a moderate external quality factor, as conceptually illustrated in Fig. 1a.

The structure of this paper is organized as follows. In Methods section, the circuit introduction and dispersion characteristic analysis of the BPSWS, mode analysis and cold-test experiments and PIC simulation results of beam-wave interaction are given. Conclusions are drawn in Results section. Some useful discussions are presented in Discussion section.

## Methods

### Circuit description and dispersion characteristic

Based on the above analysis, a strongly-coupled 2π mode is proposed by combining the relative advantages of BPSWS (π mode) and traditional 2π mode. Specifically, the BPSWS is achieved by introducing staggered long-and-short slot gratings based on the typical mono-periodic slow-wave structure (MPSWS) with long slot gratings. For a more intuitive understanding of the topological layout for the BPSWS, cross-sectional schematics of the BPSWS are shown in Fig. 1c–e.

The initial concept for generating a strongly-coupled 2π mode by utilizing a bi-periodic configuration is as follows. Essentially, the gratings of the BPSWS consist of 20 long slots and 19 short slots, as shown in Fig. 1c and d, which are aligned interleaved along the beam tunnel. For obtaining a larger input power capacity within the finite circuit area, as demonstrated in Fig. 1a, a sheet electron beam is utilized which traverses through the central region of the BPSWS gratings. Further, a pair of superdimensional longitudinal overmoded coupling cavities are symmetrically positioned along the y-axis of the BPSWS, facilitating the extraction of longitudinal power. Additionally, the electric field intensity pattern revealed in Fig. 1b denotes the concurrent presence of features characteristic of a typical longitudinal TM_31_ overmoded configuration within the BPSWS: the envelope delineates three distinct portions along the y-axis. As for the longitudinal (z-direction) electric field distribution illustrated in Figure (b), it distinctly signifies the attributes of the 2π mode: each long slot is inhabited by a potent wave envelope, and there should be adequate isolation between these envelopes to deter the in-tunnel propagation for TM waves. The dynamics of the ‘strong-coupling’ mechanism will be delineated in “Mode analysis” section.

**Figure 2:**
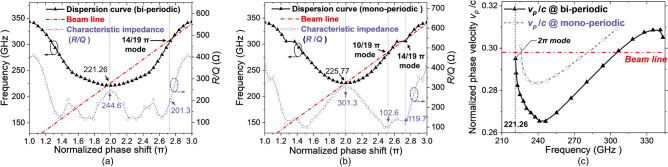
Dispersion curves and *R/Q* plots corresponding to the normalized phase in (**a**) BPSWS and (**b**) MPSWS, respectively, along with (**c**) the normalized phase velocity as a function of frequency in both SWS configurations.

The high-frequency characteristics of the BPSWS were calculated using the three-dimensional electromagnetic simulation software, CST-MWS^18^. For comparison, computation results pertaining to the MPSWS are also provided. It should be noted that both the MPSWS and BPSWS, which both use a 2π-mode scheme, are designed within the same frequency band. Within this band, the lower cut-off frequencies of the passband are closely aligned, and the phase velocities are maintained at essentially equivalent levels. Such setup ensures that the low cut-off frequencies in the passband are relatively coherent, while maintaining a uniform phase velocity across the spectrum^19^. Following simulation assessments, the derived structural-and-characteristic parameters for the BPSWS, as detailed in Table 1. Additionally, the structural dimensions for the MPSWS, in line with those of the BPSWS, are clarified based on the same parameters in Table 1. With those sets of parameters, comparative calculations of dispersion and characteristic impedance were carried out, the results of which are depicted in Fig. 2a–c. The dispersion curve was determined using the resonance method for identifying specific frequency points; in the eigenmode simulation, it was set with electric boundary conditions and a background of metal with a conductivity of 2 × 10^7^ S/m. The choice of this effective conductivity is based on the additional ohmic loss effects of high-frequency fields described in the Hammerstad model^20^, corresponding to an attenuation factor of approximately 3 times.

**Table 1:** Typical structural-and-characteristic parameters for the proposed circuit.

Symbol/Quantity	Value	Symbol/Quantity	Value
*gx1*	0.81 mm	*tx*	0.19 mm
*gx2*	0.67 mm	*ty*	0.83 mm
*gy*	0.83 mm	Total SWS length	~ 8 mm
*p*	0.40 mm	Long slot number	20
*cx*	0.93 mm	Short slot number	19
*cy*	1.11 mm	Filling factor	0.79

As illustrated in Fig. 2a–c, both types of SWSs display exceedingly narrow passbands and excellent mode isolation. Additionally, they share an identical order of magnitude for the phase velocity within the common frequency band. The fundamental 2π modes for both BPSWS and MPSWS are within the 0.22 THz frequency range. The synchronization conditions between the beamline and each mode are described clearly in Fig. 2a, along with the unique characteristic impedance of each mode. This also underlines theconsiderable *R/Q* value of 244.6 Ω that the BPSWS can achieve with the 2π mode point, facilitating the design voltage operation at ~ 25 kV. By contrast, the potential parasitic 14/19π modes derived from the beamline have a modestly lower *R/Q* value of 201.3 Ω, which facilitates ensuring the priority of the 2π single-mode operation. Similarly, as depicted in Fig. 2b, the MPSWS has an *R/Q* value of 301.3 Ω in the 2π mode, which is substantially higher than the potentially parasitic 10/19π and 14/19π modes encountered in the beamline^21^. Figure 2c introduces the correlation function between the synchronised phase speed and frequency, with this providing a prerequisite for comparing the saturated output power and electronic efficiency for the BPSWS-EIO and the MPSWS-EIO.

### Mode analysis

The way to get strongly-coupled 2π modes to be established is to start with the construction of a field configuration that provides higher coupling coefficients than typical 2π mode distributions. Numerous well-proven typical 2π-mode extended interaction structures have employed MPSWS, with the 2π phase electric-field occupying each long gap. However, a more general shortcoming of these structures is that, realistically, the 2π-phase electric field will penetrate (or be called coupled) into the beam tunnel. That would still result in a reduction in the effective characteristic impedance *M*^*2*^*R/Q* and coupling coefficient *M* for the SWS, even though the beam tunnel is ideally cutoff for that frequency. Such an imperfection was previously considered to be inherent in the 2π mode under non-ideal conditions.

**Figure 3:**
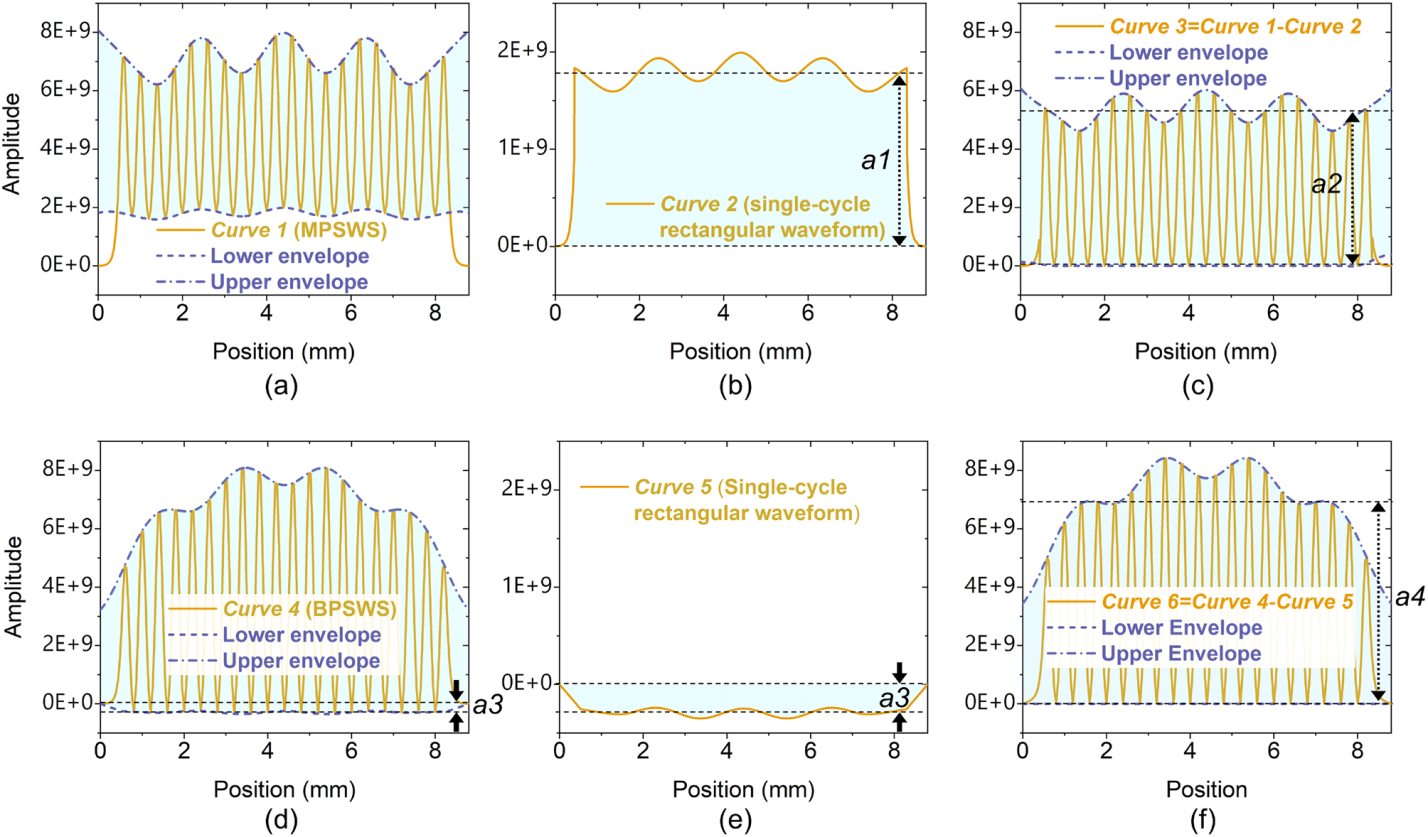
(**a**) Electric field distribution within the MPSWS, along with the amplitudes of (**b**) its square-wave and (**c**) its periodic pulse components; and (**d**) the electric field distribution curve for the BPSWS, with the amplitudes of (**e**) its square-wave and (**f**) its periodic pulse components. The energy capacity of the cavity is set at 1 J.

In an effort to provide a clearer depiction of the 2π mode electric field distribution under such non-ideal conditions, Fig. 3 presents an analysis of the electric field distribution curves along the centerline for both the BPSWS and MPSWS 2π modes. Specifically, in Fig. 3a, *Curve 1*, representative of the MPSWS, illustrates the conventional distribution pattern of the 2π mode. This pattern in MPSWS, as depicted in Fig. 3a, is essentially a simple superposition of a single-cycle rectangular waveform (illustrated in Fig. 3b, *Curve 2*) and a periodic pulse waveform (shown in Fig. 3c, *Curve 3*). The periodic pulse waveform is composed of *N* discrete envelopes, where *N* denotes the count of periods in the slow wave structure. Principally, as seen in Fig. 3b, the component of the single-cycle rectangular wave is not designed to modulate electrons; it solely functions to alternately accelerate and decelerate electrons across a designated time span. In MPSWS, the modulation of electron velocity is primarily influenced by the periodic pulse waveform components, which effectively modulate by alternating the acceleration and deceleration of electrons at varied phases, with the modulation intensity discernible through the wave amplitude.

Conversely, Fig. 3d (*Curve 4*) portrays the electric field strength distribution in BPSWS, which, similar to MPSWS, appears to be a superposition of a single-cycle rectangular waveform (as shown in Fig. 3e, *Curve 5*) and a periodic pulse waveform (demonstrated in Fig. 3f, *Curve 6*). It almost does not contain a single-cycle rectangular wave component, showing a pure periodic pulse waveform with a maximized amplitude of *a4*. The negligible negative amplitude, represented as *a3*, are nearly zero, quantified as *a3* ~ 0.13*a1 ~ 0.03*a4. The insights from Fig. 3 suggest that the prevalent issues in the 2π modes, such as the non-ideal field strength curve observed in MPSWS, could potentially be resolved by employing the 2π-mode BPSWS approach. From a velocity modulation perspective, 2π-mode BPSWS enhances the amplitude of the periodic pulse waveform component involved in the modulation process by 0.3 times, identified as *a4* ~ 1.30*a2. From a broader physical concept, this improvement primarily originates from the fact that the weak π-phase field in shorter slots negates the direct current component of the periodic pulse waveform, thereby elevating the system efficiency—a pivotal design strategy for Power Amplifier (PA) systems. Moreover, the existence of a weak π-phase field in BPSWS, being two orders of magnitude inferior to the amplitude, is essentially inconsequential, leading to inherent fundamental mode in BPSWS being considered a strongly-coupled 2π mode, rather than a π mode.

**Figure 4:**
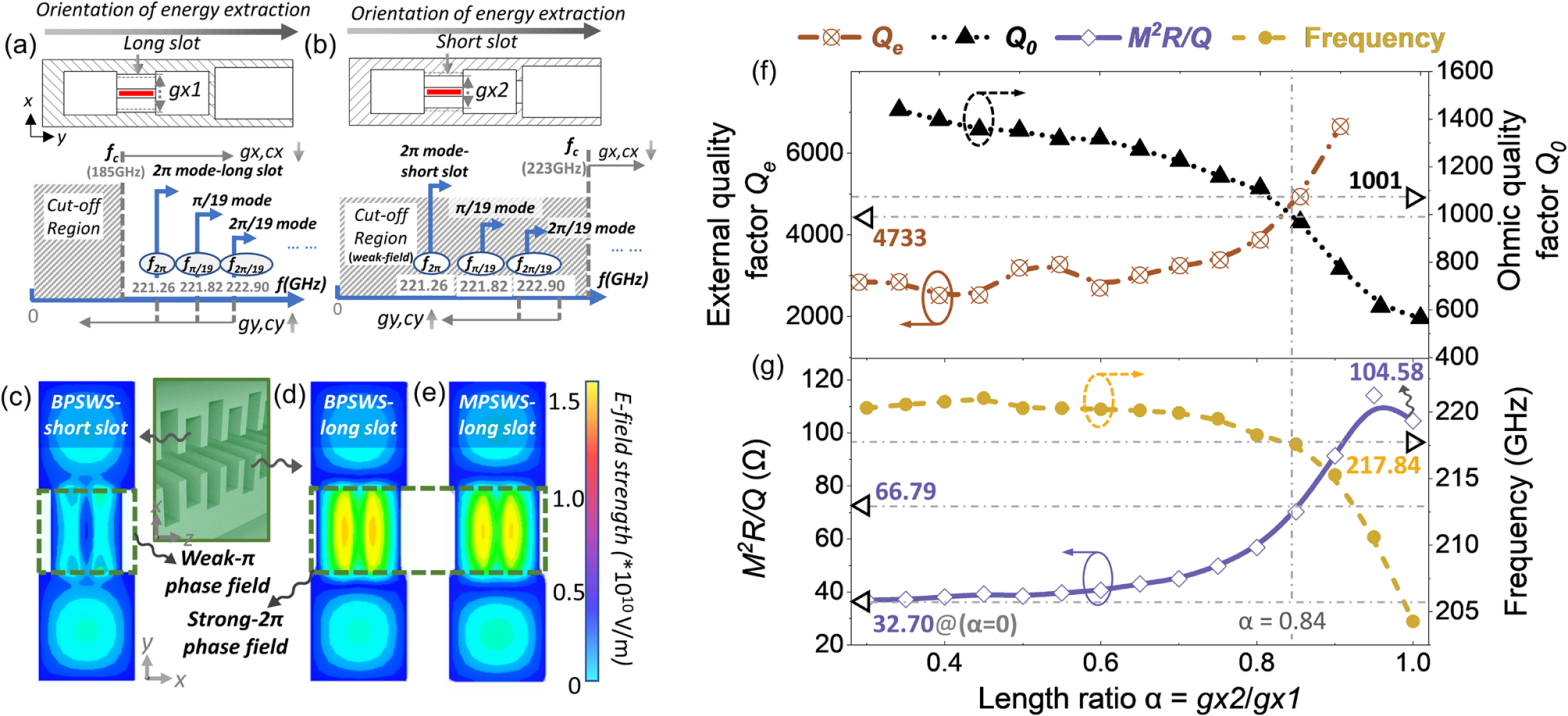
Diagrams of mode cut-off characteristics for the transversal transmission of the (**a**) long slot and (**b**) short slot. The E-field distribution contours for the (**c**) short and (**d**) long slot in BPSWS, and for the (**e**) long slot in MPSWS. High-frequency characteristic parameters (**f**) *Q*_*e*_ and *Q*_*0*_; (**g**) *M*^*2*^*R/Q* and frequency) as a function of the length ratio *α*.

To validate the feasibility of the design principle and to further explore the high-frequency characteristics for the BPSWS, the transmission mechanisms of the BPSWS were analyzed in Fig. 4a,b for the long-slot and short-slot, respectively.

Firstly, assuming that the long and short slot that support beam-wave interactions can be considered as a segment of a rectangular waveguide^22^, then all potential in-slot eigenmodes are distributed as TE_10_ waves in the interaction gaps, and thus the in-slot losses (or transmission characteristics) for those in-slot eigenmodes are determined by the gap structure. The in-slot mode cutoff characteristics are displayed in Fig. 4a,b. Compared to the potential high-frequency parasitic modes (π/19, 2π/19 modes), the electric field of the 2π mode in this long slot is more likely to be transmitted as the lower-frequency dominant mode, as shown in Fig. 4a. Viewed longitudinally along the *z*-axis, each long gap operates at a 2π phase at this point. Here, the cutoff region depends on the cutoff frequency for the long-slot rectangular waveguide. According to the mode transmission characteristics, when the *x*-axis-dimensions *gx*, *cx* decrease, the cutoff frequency increases. Whereas transmission-direction dimensions *gy*, *cy* determine the waveguide wavelength in the structure. When *gy*, *cy* increase, the guide wavelength increases, and the mode intrinsic frequency correspondingly decreases.

By the same token, Fig. 4b illustrates the mode cut-off characteristics in the short slots where the cutoff frequency exceeds the fundamental 2π mode intrinsic frequencies. When *gx2* is determined to be lower than 0.69 mm, the short slots exhibit weak transport properties. From the longitudinal view in the *z*-axis, each short slot is located between a pair of 2π electric field phases (two long slots), exhibiting a weak π-phase field. This finding corroborates the depiction presented in Fig. 2. The aforementioned details comprehensively elucidate the reasons behind the disparities observed in the rectangular wave pulses between Fig. 3b and e. The variations in the former arise from the "non-ideal" conditions encountered in conventional MPSWS, whereas the differences in the latter result from the incorporation of a weak π phase field within BPSWS.

Figure 4c–e display the contourmaps for the field distributions in *x–y* plane with the BPSWS long slots, BPSWS short slots, and the MPSWS long slots, respectively. As for the long slots, both in BPSWS and MPSWS, relatively strong interaction fields are observed since the frequencies of the 2π modes are all above the cutoff frequency and serve as the lowest operating modes.

Further to illustrate, while maintaining the long slot width *gx1* as constant, the variations of the characteristic parameters with the length ratio (*α* = *gx2/gx1*) are analyzed. As shown in Fig. 4f, the external quality factor (*Q*_*e*_) and unloaded quality factor (*Q*_*0*_) respectively exhibit a linear increase and decrease as α increases, with the *Q*_*e*_ increasement becoming particularly noticeable when α exceeds 0.85. When external circuit load impedance is matched, a greater *Q*_*e*_ may limit the circuit capacity for coupling and output. This may even lead to an internal short circuit or over-modulation, subsequently reducing output power. Even though the *M*^*2*^*R/Q* value attains 104.58 Ω when α equals 1, considering the power matching mechanism, it is advisable that α be maintained below 0.85. On the other hand, Fig. 4g shows the function of *M*^*2*^*R/Q* and resonant frequency with respect to *α*, indicating that *M*^*2*^*R/Q* and frequency monotonically increase and decrease, respectively, as *α* increases. As a key parameter of the extended interaction circuit,* M*^*2*^*R/Q* is considered to be linearly related to efficiency. After balancing the effects of *M*^*2*^*R/Q* and* Q*_*e*_, *α* is determined to be 0.84 (*gx2* = 0.67 mm), which corresponds to a *M*^*2*^*R/Q* of 66.79 Ω, and a moderate *Q*_*e*_ of 4733. Compared to the MPSWS corresponding to the case of *α* = 0 in Fig. 4g, the value of *M*^*2*^*R/Q* for the BPSWS is increased from 32.70 Ω to 66.79 Ω, which confirms the rationality for the design of the strongly-coupled 2π-mode operating mechanism.

## Results

### Cold-test experiments

**Figure 5:**
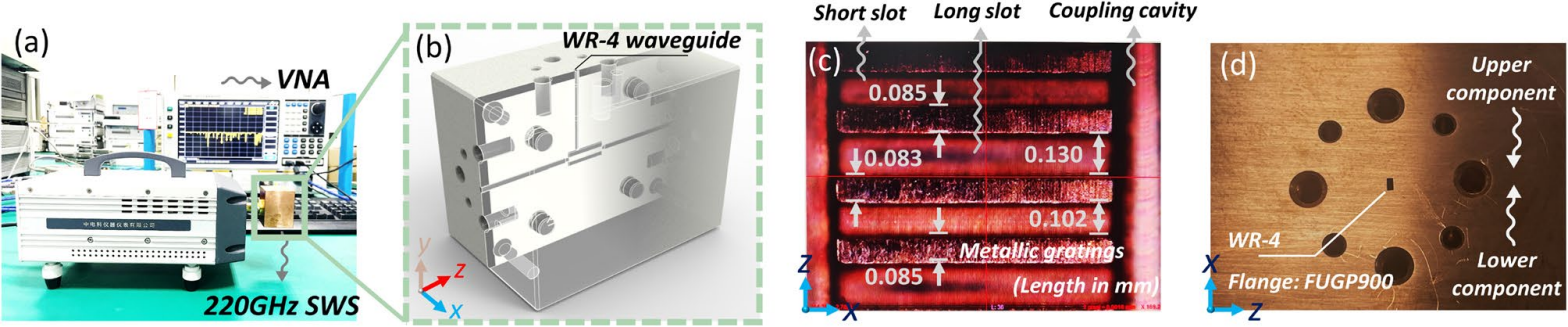
(**a**) Photograph depicting the cold-test for the structure and (**b**) the overall assembly diagram of the proposed EIO. Surface details for (**c**) the metal-gratings and (**d**) the waveguide flange section of the structure were observed, respectively.

**Figure 6:**
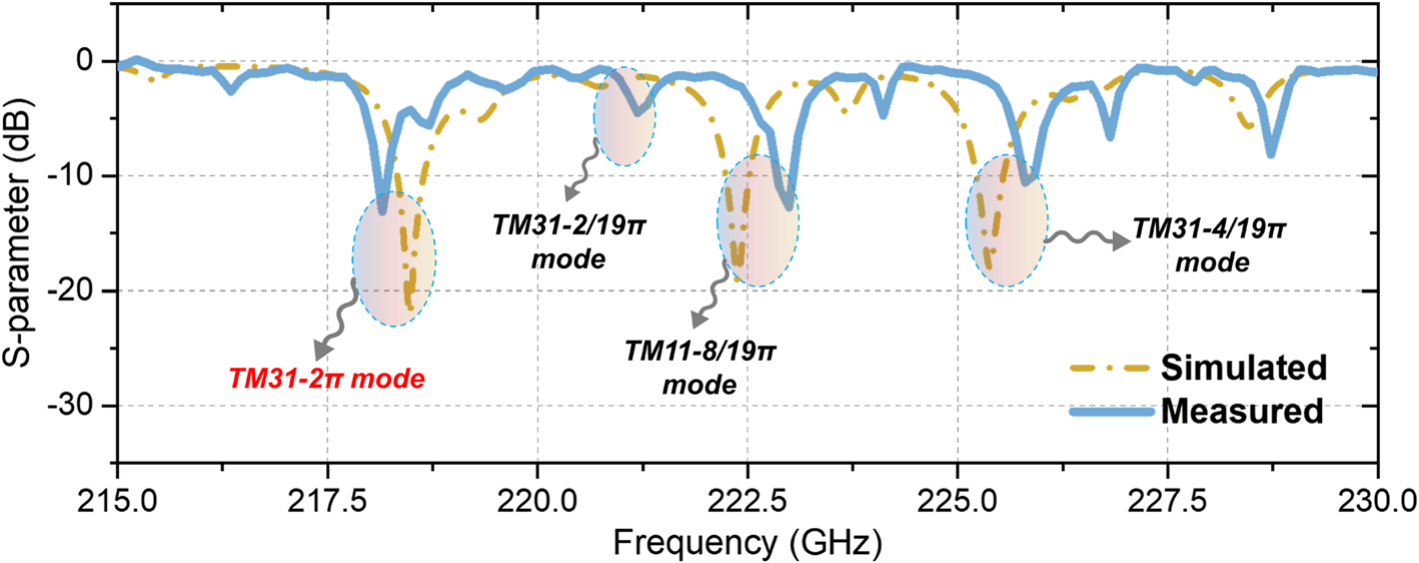
Testing and simulation profiles of S11 parameters.

The precision manufacturing for the compact 0.22 THz bi-periodic SWS could pose a challenge, which has been addressed through cold-test experiments. Figure 5a features a comprehensive photograph from the cold testing conducted with a vector network analyzer (VNA), including the detailed assembly of the complete structure shown in Fig. 5b. Figure 5c illustrates the bi-periodic metal-gratings, and Fig. 5d provides a close-up of the waveguide flange area. The results are promising, demonstrating satisfactory uniformity and precision in the CNC-manufacturing process, with a maximal fabrication error under 10 μm and deviations in metal grating size kept within 2 μm in *z*-axis. The copper structure used for the cold test has a total weight of 429 g. Figure 6 illustrates the *S11* test outcomes, which align closely with the simulation data. The test measurement for the strongly-coupled TM_31_-2π mode is approximately 330 MHz lower than the simulations, which can be attributed to manufacturing errors and chamfer issues. Additionally, the frequencies of several parasitic modes correspond well with the simulations, displaying errors generally less than 500 MHz.

### Beam-Wave interactions

**Figure 7:**
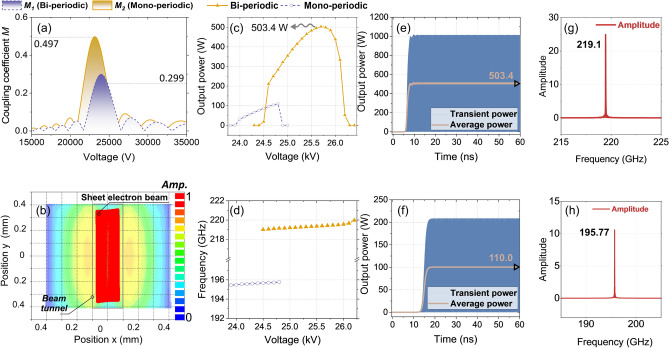
(**a**) Comparison of coupling coefficients for BPSWS and MPSWS; (**b**) electron beam profile before oscillation-starting; The voltage-dependent comparisons of (**c**) average output power and (**d**) frequency for both SWSs; The time-dependent waveforms of transient power and average power for both (**e**) BPSWS and (**f**) MPSWS, and the spectral analyses corresponding to (**g**) BPSWS and (**h**) MPSWS, respectively.

Extensive 3D simulations are carried out for the proposed BPSWS-supported strongly-coupled 2π mode. The coupling coefficient is recognized as an essential variable to characterize this BPSWS, which represents the ratio of the actual modulation voltage received by the electron beam to the voltage loaded on the SWS. Thus, the coupling performance of the strongly-coupled 2π mode is initially examined and contrasted with the traditional 2π mode supported by MPSWS. As depicted in Fig. 7a, this comparison shows the coupling coefficients as a function of voltage for both MPSWS and BPSWS. The results reveal a significant enhancement in the coupling coefficient for the MPSWS-based BPSWS from 0.299 to 0.497, showing a notable improvement of 66.2% than MPSWS. From a physical perspective of *M*, this improves the beam-wave transduction efficiency per unit length.

Furthermore, Fig. 7b also took into consideration the beam geometry towards the end of the BPSWS to ensure that the particle beam flow rate was not interfered by the Diocotron instability^23^ and Brillouin thermal-beam spreading; a common set for magnetic field strength of 0.8 T was chosen, corresponding to an electron transmission rate of 100%, with no electrons being intercepted. The uniform magnetic field of 0.8 T can be accomplished by arranging Neodymium-Iron-Boron (NdFeB) permanent magnets, as demonstrated in the G-band magnetic focusing configuration^24^ by Qu et al.

The self-oscillation process of electromagnetic signals was simulated via CST Particle Studio. The SWS circuit is composed of oxygen-free copper with a conductivity of 2 × 10^7^ S/m, considering the distributed losses. Operating at an optimal voltage of 25.8 kV, the circuit uses a beam current of 0.3 A (equating to a current density of 277 A/cm^2^). The dimensions of the sheet beam are 0.83 × 0.19 mm, with a filling factor of 0.79 along the x-axis, where the filling factor represents the ratio of the beam tunnel size to the electron beam size.

Both the saturated power and the resonant frequency, as a function of beam voltage, for the BPSWS-EIO and MPSWS-EIO can be derived from Fig. 7c and d, respectively. As illustrated in Fig. 7c, the BPSWS-EIO delivers stable average output power over a voltage range of 24.7–26.1 kV, peaking at approximately 503.4 W at 25.7 kV (for a sinusoidal signal, output power *P*_average_ = *P*_peak_/2) with a frequency of 219.1 GHz. While the MPSWS-EIO delivers stable output over the 24.0–24.8 kV range, with ~ 110W output power and 195.77 GHz frequency at 24.8 kV, as shown in Fig. 7d. The results from the PIC simulations that the synchronous beam voltage during the beam-wave interaction aligns closely with the ~ 25 kV synchronous voltage depicted in Fig. 2a–c, thereby validating the efficacy of the proposed design. Figure 7e and f represent the transient/average output power plots for both BPSWS-EIO and MPSWS-EIO at 25.7 kV and 24.8 kV, respectively. The PIC simulations prove stable operations for both EIOs, with the optimal efficiencies reaching 6.52% for BPSWS-EIO and 1.48% for MPSWS-EIO, as shown in Fig. 7e and f. Accordingly, the output signals from the two EIOs were subjected to Fourier transform analysis, resulting in frequency spectra that are clean and devoid of noise, as depicted in Fig. 7g and h. These findings confirm that the proposed BPSWS and MPSWS are both capable of achieving effective beam-wave interaction performance, with the strongly coupled 2π mode showing a significant enhancement of approximately 5%. At this point, the MPSWS is operating at a non-desirable hetero mode at 195.77 GHz, while the desired 2π mode at 0.22 THz fails to be excited. The improvement in efficiency mentioned above results from the collective influence of factors including *M*^*2*^*R/Q*, the *Q*-factors, and the length of the circuit, among others. Therefore, it does not exhibit a linear relationship with any individual parameter.

In conclusion, our comprehensive investigation centers around a novel strongly-coupled 2π mode BPSWS, which evolves from the conventional MPSWS, interacting with a sheet electron beam. The new BPSWS reveals a prominent attribute of possessing a highly effective characteristic impedance and wave amplitude (22% improvement over conventional MPSWS) within a relatively concise interaction distance. Simultaneously, in contrast to MPSWS-EIO, the proposed BPSWS-EIO significantly enhances both the output power (3.57 times improvement) and electronic efficiency (5% improvement).

Benefiting from a *M*^*2*^*R/Q* value of up to 66.96 Ω, the sheet-beam-based BPSWS-EIO is expected to achieve an average output power exceeding 250 W and an electronic efficiency surpassing 3.3% within the voltage tuning scope of 24.7–26.1 kV at 0.22 THz band. Specifically, at 25.7 kV, the maximum electronic efficiency, saturated power, and frequency are 6.52%, 503 W, and 219.42 GHz, respectively.

This suggests that the proposed BPSWS-supported, strongly-coupled 2π mode demonstrates considerable promise as an operational mode. Notably, it holds the potential to maintain increased efficiency in millimeter-wave to terahertz SWSs over a specified circuit length.

## Discussions

Considering the present limitations in achieving optimal efficiency within the available 0.22 THz SWSs, typically below 5% due to significant ohmic losses and related factors at THz frequencies, it is important to clarify the following points.

For metallic terahertz SWS, the primary challenge arises from the skin depth and surface roughness being comparable, leading to considerable conducting-wall current losses. As analyzed using the Hammerstad-Bekkadal model^20^, the wall current losses attributed to the copper-surface roughness can effectively reduce the conductivity. Following the Hammerstad model, a cautious estimate of conductivity has been set at 2 × 10^7^ S/m, corresponding to a surface roughness of ~ 0.12 μm. Further, cold testing has proven the SWS presented in this paper to be feasible and replicable, with electron beam parameters within the practical ranges established in previous research. This approach is considered to provide a reasonable estimation of the maximum efficiency attainable with the proposed SWS.

To the best of the authors’ knowledge, in the ~ 0.2 THz band of EIDs, CPI is at the forefront, having achieved promising output power and efficiency performance. A 198 GHz EIA reported by CPI in 2023 achieved a power output of 140W and an efficiency of approximately 4.4%, operating at a 5% duty cycle, though the specifics of the slow-wave structure utilized remain undisclosed^25^. In the same year, AIRI-CAS unveiled a 220 GHz EIA, achieving outputs of 195W in simulations and 120W in experiments, respectively, yielding an electronic efficiency of ~ 3.0%^24^. From a simulation perspective, for instance, a sheet-beam G-band EIA reported an output of about 500W with a 3.7% efficiency^26^; a high-efficiency G-band EIA at 220 GHz reached an efficiency of 7.9%^27^, but the six-cavity structure used significantly extended the interaction distance, estimated to be ~ 21 mm in total length, thereby limiting the gain per unit length. Reports of high-efficiency EIOs in the G-band are still scarce, with most efficiencies below 4%^28–30^. In this study, a single-cavity, 39-gap EIO structure is utilized, attaining an electronic efficiency of 6.52% within an interaction length of approximately 8 mm. The brief parameters for the above devices are given in Table 2. Viewed from the simulation-based perspective, this design shows a higher interaction efficiency relative to current EIO research and offers a significant advantage in compactness when compared to existing EIAs. The relatively high interaction efficiency demonstration also validates the effective application for the proposed SWS in enhancing the transition between π and 2π modes.

**Table 2:** A few typical G-band EIO performance metrics for this stage.

Ref	Device	Power/Efficiency	Total SWS length
^24^	EIA	195W/ ~ 4.9%	~ 12 mm
^25^	EIA	140W/ ~ 4.4%	–
^26^	EIA	500W/ ~ 3.7%	~ 15 mm
^27^	EIA	390W/7.9%	~ 14.4 mm
^28^	EIO	503W/ ~ 0.94%	~ 5 mm
^29^	EIO	2.1 kW/ ~ 3.4%	~ 5.5 mm
This paper	EIO	503W/6.5%	~ 8 mm

## Data Availability

The data that support the findings of this study are available from the corresponding author upon reasonable request.
